# Detection of Subclinical Neurotrophic Keratopathy by Non-Contact Esthesiometry

**DOI:** 10.21203/rs.3.rs-2833826/v1

**Published:** 2023-05-22

**Authors:** Marta Villalba, Victor Sabates, Seyyedehfatemeh Ghalibafan, Victor Perez, Swarup Swaminathan, Alfonso Sabater

**Affiliations:** Duke University School of Medicine, Durham, NC; Duke University School of Medicine, Durham, NC; Duke University School of Medicine, Durham, NC; Duke University School of Medicine, Durham, NC; Bascom Palmer Eye Institute; Bascom Palmer Eye Institute

## Abstract

**Objectives.:**

To analyze corneal sensitivity with a new noncontact and hand-held esthesiometer (Brill Engines, Spain) in patients with dry eye disease (DED) and patients on hypotensive drops, and to compare it with healthy subjects.

**Methods.:**

31 patients (57 eyes) with DED, 23 patients (46 eyes) with glaucoma and 21 healthy patients (33 eyes) were recruited. In all patients, corneal sensitivity was measured. Subsequently, a keratography test (Keratograph 5M, Oculus) was carried out to measure tear meniscus height (TMH), non-invasive break up time (NIBUT), bulbar redness (Jenvis scale) and corneal staining (CS, Oxford scale). Both corneal sensitivity and ocular surface parameters were compared between DED, glaucoma, and healthy subjects. Linear mixed models were constructed to utilize data from both eyes of patients. A 95% confidence level was considered statistically significant.

**Results.:**

The mean age was 56.1±16.1 years in DED group, 69.5±11.7 years in the glaucoma group and 36.3±10.5 years in the control group. Adjusting for age and sex, esthesiometry was significantly worse in DED and glaucoma vs control group (p = 0.02 and p = 0.009, respectively). NIBUT was lower in DED and glaucoma patients (p < 0.001 and p = 0.001, respectively). Redness and CS values were higher in DED group (p = 0.04 and p = 0.001, respectively). TMH was lower in the glaucoma patients (p = 0.03).

**Conclusions.:**

Corneal sensitivity measured with a novel noncontact esthesiometer was reduced in DED and glaucoma patients compared to controls. In clinical practice, this esthesiometer could be an easy-to-use device to evaluate for patients with subclinical neurotrophic keratopathy.

## INTRODUCTION

Dry eye syndrome is one of the most frequent ocular surface diseases (OSD), and is characterized by a loss of tear film homeostasis followed by ocular symptoms. Inflammation and damage to the ocular surface, neurosensory abnormalities, tear film instability, and hyperosmolarity all play etiological roles (1). Another frequent cause of OSD is the use of topical eye drops, such as antibiotics or other medications (2). In the case of glaucoma patients, topical medication is often first-line therapy; they are frequently used for decades, often causing dry eye disease (DED) symptoms and corneal damage (3). Some of the most used, timolol and latanoprost, have been shown to cause adverse effects such as decreased tear production and keratitis, and even inhibition of epithelial cell proliferation (4, 5). The preservatives contained in these eye drops also cause corneal irritation (6). Due to pain and visual impairment, DED and OSD may affect the quality of life of the patients (7). Dryness is the most common symptom, followed by ocular fatigue, grittiness, redness, and discomfort (8).

The stimulation of sensory nerves at the ocular surface may explain why symptoms are present (9). However, there is often a lack of correlation between objective exam findings and subjective patient symptoms (10). The cornea is the human tissue with the highest density of sensory and autonomic nerve fibers (11, 12). Different studies have demonstrated the existence of three different types of sensory receptors: mechanoreceptors, thermoreceptors and polymodal receptors that detect mechanical, thermal, and chemical stimuli respectively (13, 14). Additionally, corneal nerves provide important trophic supply to corneal epithelium and contribute to the maintenance of a healthy ocular surface (11, 12, 15). Tear instability and epithelial damage can disrupt corneal epithelial barrier function (16), which permits greater access to adverse environmental conditions (air, low humidity) to the corneal sensory receptors and makes the cornea more vulnerable to injury (17). Prolonged erosion and irritation of the ocular surface may elevate the pain threshold, resulting in reduced corneal sensitivity (15, 18, 19) and long-term dysfunction of corneal innervation, a degenerative condition known as neurotrophic keratitis (20, 21).

Various methodologies have been utilized to evaluate corneal nerves. *In vivo* confocal microscopy (IVCM) can be used to observe and evaluate morphology and number of corneal nerves (22), but the method of assessing nerve function is by measuring corneal sensitivity. In cases of corneal damage after refractive surgery and when using contact lenses (CL), the evaluation of ocular surface sensitivity is a helpful indication of corneal physiology (23, 24). Cochet-Bonnet is the most used esthesiometer in research and clinic (25, 26). It employs a nylon thread, the length of which may be modified to control the force applied to the cornea and assess corneal sensation. However, it has certain drawbacks, including the fact that it is invasive, the force delivered is nonlinear, and the precise position and force of the stimuli are poorly replicated (27, 28). Different prototypes of esthesiometers were developed to correct the shortcomings of Cochet-Bonnet. Most of them are noncontact esthesiometers and employ pulses of compressed air (29, 30, 31, 32). They produce thermal (33, 34), chemical (35), mechanical or a combination of stimuli (29, 30), which target different nociceptors in a preferred manner. One of the best known is Belmonte noncontact esthesiometer, which allows users to alter the flow, temperature, and CO2 concentration of the gas jet, and provide various stimuli for the ocular surface (32, 36, 37). According to published studies, this esthesiometer can evaluate ocular surface sensitivity in a repeatable manner (38).

The purpose of the study was to analyze corneal sensitivity with a new noncontact and hand-held esthesiometer (Brill Engines esthesiometer) (39, 40) in patients with DED signs and/or symptoms, as well as patients receiving IOP-lowering therapy. This esthesiometer is a sensitive, non-invasive, portable and easy-to-use device to test corneal sensitivity. We hypothesize that this test may allow the identification of patients whose corneal nerves are subclinically damaged, allowing the physician to implement early therapeutic decisions (e.g., consideration of glaucoma surgical intervention) and avoid long-term irreversible corneal damage.

## MATERIALS AND METHODS

### STUDY POPULATION

This study was approved by the Institutional Review Board of the University of Miami Miller School of Medicine. The protocol conformed to the requirements of the United States Health Insurance Portability and Accountability Act and the tenets of the Declaration of Helsinki. The DED group in the study were those with Ocular Surface Disease Index (OSDI) score ≥ 13 and/or signs of DED, corneal staining (CS) ≥ 2 and/or non-invasive break up time (NIBUT) average < 10. The calculation of these metrics is discussed below. The glaucoma group included those who use or used at any time in the past ocular hypotensive medications. Finally, the control group was comprised of healthy patients who were asymptomatic with no signs or symptoms of dry eye and did not use any type of eye drops or topical medication that may affect the ocular surface. Exclusion criteria for both groups included pregnancy and ages less than 21 or over 90 years. Patients in the study were evaluated at the Bascom Palmer Eye Institute and informed consent was obtained from all of them prior to clinical examination. All patients were asked about ocular pathologies, use of contact lenses or eye drops, and previous ophthalmologic surgeries.

### OCULAR SURFACE DISEASE INDEX (OSDI)

Ocular symptoms were evaluated using the OSDI test before performing the rest of the clinical examinations. It is the most used questionnaire for determining symptom severity in ocular surface disease patients. It is comprised of 12 questions about the signs and symptoms of dry eye, how they affect vision, the restrictions they bring about, and environmental variables that may contribute to dry eye. Each question is given a score between 0 and 4. The values are then summed, and a subject with a score ≥ 13 was considered symptomatic.

### ESTHESIOMETRY

The corneal sensitivity was measured using a non-contact esthesiometer (Brill Engines, Spain). This device produces pulses of air at different intensities, with five levels and a pressure range of 2 mbar to 10 mbar. Each pressure range is defined as the average estimated pressure over a 0.4 mm diameter surface which is 4 mm distant from the outlet nozzle. To ensure that the assessment is performed at the correct distance, it has a camera and an electronic positioning system. The test was performed by placing the device on the slit lamp. Three measurements at each level were taken, in the lower quadrant of the cornea, starting at the lowest level (level 1–2mbar) and increasing it until the patient sensed the air puff. The lowest level that the patient could feel the air was recorded. In patients who denied any sensation, the value was recorded as 11 mbar, one mbar above the maximum value of the instrument.

### KERATOGRAPHY

The Oculus Keratograph 5M Topographer (Oculus Optikgeräte GmbH, Wetzlar, Germany) was used for the examination of the ocular surface. The following parameters were measured: tear meniscus height (TMH; measured in mm), average non-invasive break up time (NIBUT; measured in seconds), and bulbar redness (JENVIS grading scale). After applying topical fluorescein, CS was determined, using the OXFORD Scheme grading scale (41).

### STATISTICAL ANALYSIS

Statistical analysis was performed using R 4.2.2 (R Core Team, Vienna, Austria). Analyses were completed using linear mixed models with random effects placed at the patient level to account for inter-eye correlations as previously completed (42). Data were centered on mean age of the total population, and models adjusted for both age and gender. Outcome variables that were analyzed were esthesiometry value, NIBUT, TMH, redness and CS. DED, glaucoma, and control groups were compared. An alpha level of 0.05 was used to evaluate statistical significance.

## RESULTS

### DEMOGRAPHIC CHARACTERISTICS AND CLINICAL PARAMETERS

**Figure 1:**
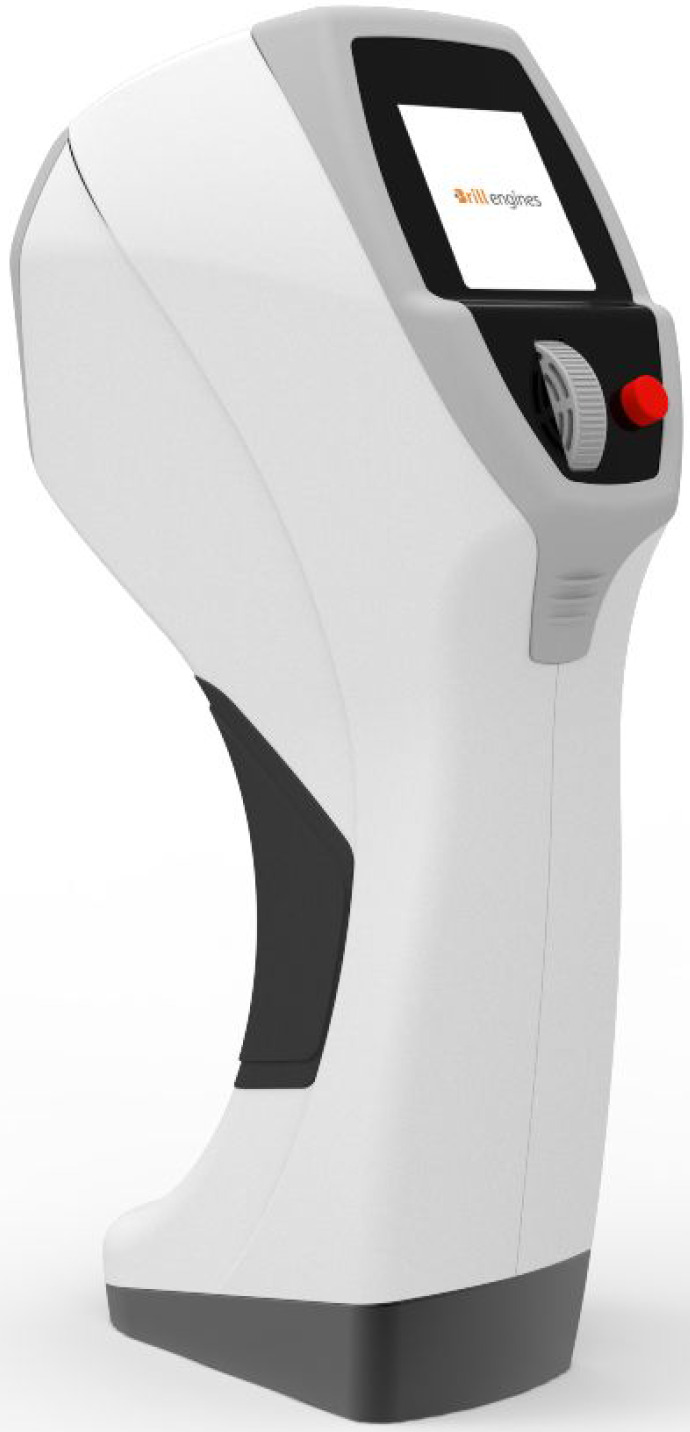
Portable and non-invasive Corneal Esthesiometer (Brill Engines, Spain) for corneal sensitivity assessment through controlled air pulses as stimuli.

This study evaluated a total of 136 eyes from 75 subjects. A total of 57 eyes from 31 subjects were in the DED group, 46 eyes from 23 subjects were in the glaucoma group, and 33 eyes from 21 subjects were controls. Demographic data are provided in Table 1. There was a statistically significant difference in age between the three groups. Data on topical medication use are listed in Table 2. Ocular surface parameters measured are provided in Table 3. Overall, the DED group had the most severe signs of ocular surface damage, with 13 (41.9%) eyes having a CS ≥ 3 (Fig. 1).

### COMPARISON OF CORNEAL SENSITIVITY LEVELS BETWEEN CONTROLS, DRY EYE DISEASE, AND GLAUCOMA GROUPS

**Figure 2:**
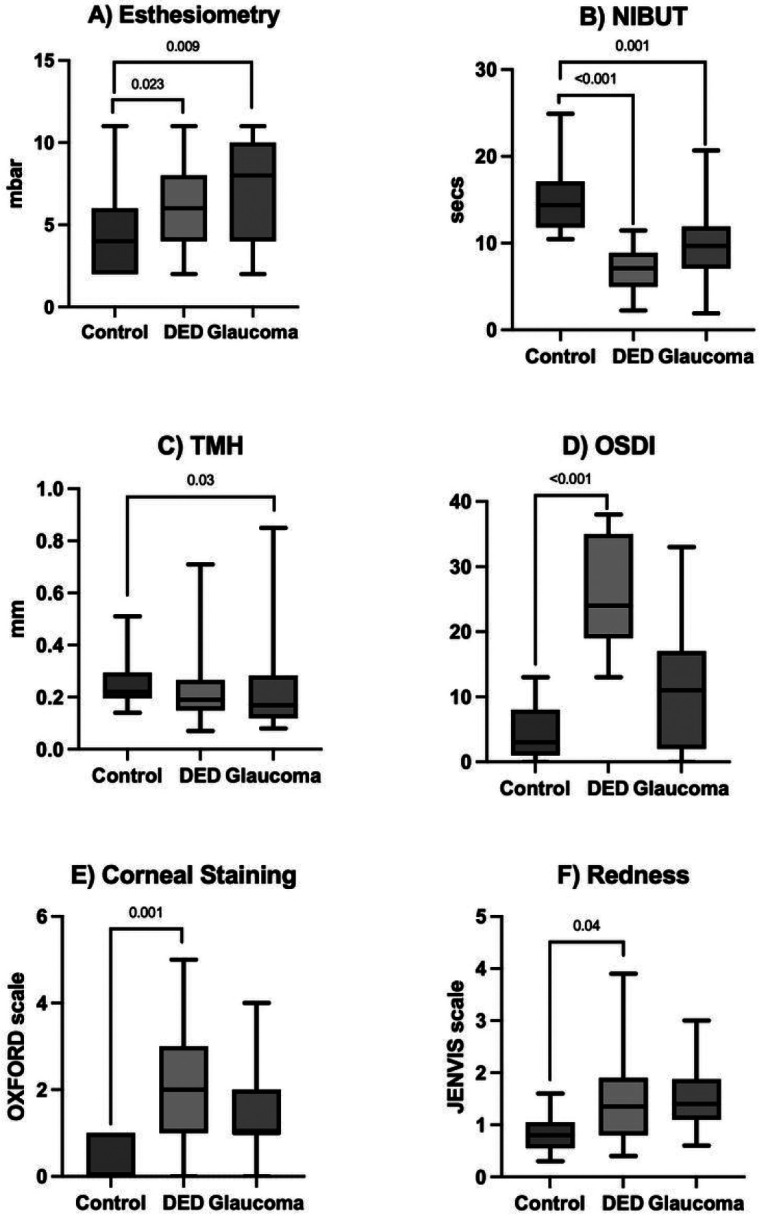
Ocular surface parameters in patients with dry eye disease (DED), glaucoma and healthy controls. A) Noncontact esthesiometry was significantly worse in DED and glaucoma patients when compared with the controls. B) Noninvasive tear breakup time (NIBUT) scores were significantly lower in the DED and glaucoma groups. C) Tear meniscus height (TMH) was significantly lower in glaucoma patients compared with the controls. D) Ocular Surface Disease Index (OSDI) scores were significantly worse in the DED group. E) The DED patients showed a significant higher value of corneal staining (OXFORD scale). F) Conjunctival redness (JENVIS scale) scores were significantly higher in DED group when compared with the healthy controls. Statistical significance was derived from linear mixed models adjusting for age and sex. Data showed as boxplots.

Given differences in demographics between groups could impact outcome variables, linear mixed models adjusting for age and sex were completed to compare corneal sensitivity levels among the study groups. With random effects placed at the patient level, data from both eyes of a patient could be utilized. Esthesiometry levels were significantly reduced in eyes receiving topical hypotensive medications, followed by the DED and control eyes (7.5±3, 6±3 and 3.2±1 mbar, respectively) with significant differences observed between the glaucoma and DED eyes when compared to controls (*P* = 0.009 and *P* = 0.023, respectively (Fig. 2).

### RELATIONSHIPS BETWEEN CORNEAL SENSITIVITY LEVELS AND TOPICAL HYPOTENSIVE MEDICATIONS

Relationships between the number of daily IOP-lowering drop applications and topical hypotensive medications with corneal sensitivity levels were examined. Corneal sensitivity levels in eyes that receive ≥ 3 administrations of IOP-lowering hypotensive eyedrops daily were significantly lower compared to eyes receiving 2 or fewer applications of hypotensive eyedrops daily (*P* = 0.04). When evaluating the relationship between corneal sensitivity and the total number of IOP-lowering medications that an eye was receiving, there was a trend towards lower corneal sensitivity in those eyes on ≥ 3 IOP-lowering medications but no statistical significance (*P* = 0.059).

### COMPARISON OF OCULAR SURFACE PARAMETERS BETWEEN CONTROLS, DRY EYE DISEASE, AND GLAUCOMA GROUPS

NIBUT was significantly reduced in DED and patients using IOP-lowering medications, with significant differences observed (*P* < 0.001 and *P* = 0.001, respectively; Table 3) versus the control patients. Redness and CS were significantly higher in the DED group (*P* = 0.04 and *P* = 0.001 respectively), but not in the glaucoma group (*P* = 0.35 and *P* = 0.09 respectively). TMH was significantly lower in glaucoma eyes compared to controls (P = 0.03). OSDI score was significantly higher in DED group, but in not the glaucoma group, compared to controls (P < 0.001 and P = 0.13, respectively).

## DISCUSSION

In this study, corneal sensitivity was measured with a new noncontact esthesiometer, demonstrating that it is reduced in both DED patients and those using ocular hypotensive drops, compared to healthy controls. It is known that corneal damage caused by ocular surface diseases or topical medications may produce damage to the corneal nerve endings (6, 9, 10, 12). This may develop an irreversible neurotrophic keratopathy if the factors that damage the cornea are not modified (20, 21). For this reason, corneal esthesiometry screening may play a crucial role in this patient population.

Corneal sensitivity has already been evaluated in other studies but with other devices. The most commonly used is Cochet-Bonnet, which tests predominantly mechanoreceptors using a nylon thread whose length can be modified (25, 26). The first difference between the Brill device and Cochet-Bonnet esthesiometer, is that the former uses an air puff instead of mechanical contact, so it may be stimulating different corneal nociceptors (mechanoreceptors, thermoreceptors and polymodal receptors). For this reason, the results obtained with this esthesiometer cannot be compared with those obtained with Cochet-Bonnet or other mechanical esthesiometers. Other authors report the use of different noncontact esthesiometers (29-37) that can measure thermal, chemical or mechanical sensitivity using compressed air or gas. However, these devices are difficult to use in daily practice. In contrast, Brill’s non-contact esthesiometer is portable, minimally invasive and easy to use by physicians, optometrist or ophthalmic technicians.

In our study there were statistically significant differences in age and sex distributions between the three groups. The ocular surface parameters measured with keratography (Keratograph 5M, Oculus) were NIBUT, TMH, conjunctival redness and CS. NIBUT was significantly lower in the DED and glaucoma group compared to controls, as shown in other previous studies (43, 44). The TMH was significantly lower in the glaucoma group. This result is the same obtained by other groups (43, 45). Compared to healthy patients, conjunctival redness and CS was also higher in both DED and glaucoma patients but was statistically significant only in the DED group.

DED eyes had significantly higher OSDI test scores compared to controls. It is interesting how in these patients, symptoms are greater than in healthy subjects despite having decreased corneal sensitivity. This has already been shown in some studies that have observed that there is no correlation between symptoms and signs in patients with OSD (7, 8). But on the other hand, the OSDI score was lower in the glaucoma group than in the DED group. The reason could be a greater damage of the corneal nerves in these patients, demonstrated by the fact that they are the group with the most reduced corneal sensitivity. This demonstrates the importance of screening these patients, since despite having damaged corneas, they do not present the same ocular symptoms as patients with DED.

Corneal sensitivity measured with this novel noncontact esthesiometer was reduced in DED patients compared to controls. This finding is consistent with prior studies in which esthesiometry was analyzed (15, 29, 46, 47). In contrast, other studies found that DED patients had significantly higher sensitivity than controls (48). This may be due to corneal nerves present in corneal epithelium are more exposed secondary to corneal injury and instability of the tear film, so they perceive sensations more intensely. Is possible that corneal sensitivity might be higher in the early stages of the disease, while it may decrease in the chronic stage. The nerves endings suffer damage by continued irritation of the ocular surface, that together with local inflammation and the inflammatory mediators that are released, cause the destruction and loss of the ability to perceive sensations in the cornea (9, 12).

Sensitivity was also significantly reduced in glaucoma patients who used ocular hypotensive eye drops, compared to dry eye patients and controls, which has been reported previously by other groups (18, 19). It is known that many patients using these medications present with OSD (6, 10). Prolonged use of these eye drops is thought to induce neurotrophic keratopathy, affecting the corneal sensitivity of patients, and increasing their risk of developing epithelial damage and ulcers that can become complicated. The damage caused by these medications is due both to the presence of preservatives (2, 3, 6) and to the adverse effects of the antiglaucoma medications themselves. For example, timolol has been shown in previous studies to have an anesthetic effect on the corneal surface (49, 50), and the proinflammatory effect of prostaglandins is a well-known adverse effect of these medications (5).

Non-contact corneal esthesiometry may be an excellent diagnostic tool, which can be easily performed by any ophthalmic technician, to determine when corneal sensitivity starts to decline in patients using topical glaucoma medications. At this point, it may be appropriate to switch these patients to preservative-free hypotensive medications, perform laser trabeculoplasty or consider surgical intervention to eliminate or reduce the number of glaucoma medications. This strategy may avoid causing an irreversible neurotrophic keratopathy (51).

This study also evaluated whether corneal sensitivity levels had a relationship with topical medications used in the glaucoma group. For the analysis we have utilized two different metrics, the number of drops applications daily and the number of different IOP-lowering medications. For example, a patient on timolol twice daily and latanoprost nightly would have 3 drop administrations daily but would only be on two medications. On one hand, the relationship between the number of daily drop instillations and corneal sensitivity showed a significant reduction in those who used ≥ 3 IOP-lowering drops per day. On the other hand, when examining the relationship between the number of medications used and corneal sensitivity, a tendency to lower sensitivity was observed in patients using ≥ 3 different medications, but it was not statistically significant. These differences in results may be because the use of a greater number of drops per day also involves a greater application of preservatives, which have been associated with corneal nerve damage in glaucoma patients (52). Therefore, corneal damage may be more related to the preservatives than to the active ingredients themselves, as reported in other studies, in which patients treated with preservative free IOP-lowering drops had less damage to the ocular surface (6, 10).

There are various limitations to this study. First, sensitivity testing is subjective because the patient determines whether they perceive the stimulus or not. The differences between esthesiometry levels are minimal and sometimes patients did not perceive the air puff, but when repeating the measurement with the same pressure they did feel it. For this reason, the measurement was repeated three times at each level. Second, patients in this study were not separated based on the type or duration of the hypotensive eye drops they were using. Further studies will be required to elucidate this aspect.

## CONCLUSION

Despite its limitations, to our knowledge this study is the first to evaluate corneal sensitivity in patients with dry eye disease and patients using glaucoma medications using Brill’s non-contact air esthesiometer. In clinical practice, this esthesiometer is a portable and easy to use screening device to evaluate corneal sensitivity in dry eye disease and glaucoma patients. This may allow to detect early stages of neurotrophic keratopathy and, consequently, implement therapeutic actions to avoid long-term damage of corneal nerves.

## Figures and Tables

**Table 1.** Demographic characteristics of the population

	Control, N = 21^1^	DED, N = 31^1^	Glaucoma, N = 23^1^	p-value^2^
**Age**	37.190 ± 11.677	56.129 ± 16.058	69.478 ± 11.797	<0.001
**Sex**				0.13
**Female**	13 (61.9%)	25 (80.6%)	13 (56.5%)	
**Male**	8 (38.1%)	6 (19.4%)	10 (43.5%)	

1 Mean ± SD; n (%)

2 Kruskal-Wallis rank sum test; Pearson’s Chi-squared test

**Abbreviations:** DED = dry eye disease

**Table 2.** Ocular topical medication (per individual eye)

Medication	Control, N = 33 eyes	DED, N = 57 eyes	Glaucoma, N = 46 eyes
**Artificial tears** ^1^	3 (9%)	44 (77.2%)	22 (47.8%)
**Autologous serum** ^1^	0 (0%)	7 (12.2%)	6 (13%)
**Topical anti-inflammatories** ^1^	0 (0%)	15 (26.3%)	10 (21.7%)
**Hypotensive medications** ^1^			
**Beta-blockers**	0 (0%)	0 (0%)	25 (54.3%)
**Prostaglandins**	0 (0%)	0 (0%)	27 (58.7%)
**Carbonic anhydrase inhibitors**	0 (0%)	0 (0%)	16 (34.8%)
**Alpha agonists**	0 (0%)	0 (0%)	8 (17.4%)
**Combination**	0 (0%)	0 (0%)	13 (28.3%)

1 n (%)

**Abbreviations:** DED = dry eye disease

**Table 3.** Coefficients from multivariable linear mixed effects model estimating various corneal parameters.

	Esthesiometry (mbar)	p	NIBUT (sec)	p	TMH (mm)	p	Redness (JENVIS scale)	p	CS (Oxford scale)	p	OSDI	p
Intercept	3,79	-	14,34	-	0,32	-	0,99	-	0,78	-	5,31	-
Age (per decade)	0,40	0,08	−0,36	0,21	0,04	0,005	0,15	0,001	0,16	0,06	0,56	0,36
Sex (male)	0,51	0,44	0,32	0,69	0,01	0,79	0,24	0,06	−0,28	0,26	−0,19	0,91
Group												
Control	-		-		-		-		-		-	
DED	2,00	0,023	−7,58	<0.001	−0,08	0,07	0,35	0,04	1,15	0,001	17,24	<0.001
Glaucoma	2,89	0,009	−4,74	0,001	−0,13	0,03	0,20	0,35	0,67	0,09	4,3	0,13

Abbreviations: NIBUT = non-invasive breakup time, TMH = tear meniscus height, CS = corneal staining, OSDI = ocular surface disease index, DED = dry eye disease
